# Evaluation of Interventions and the Impact of Pharmacist Consultations on the Management of Minor Ailments in Brazil

**DOI:** 10.1111/jep.70726

**Published:** 2026-11-09

**Authors:** Letícia Isabella Ferreira Brandão, Gustavo Pereira Calado, Laura Beatriz Gomes Brandão, Maria Christina dos Santos Verdam, Débora Santos Lula Barros, Rodrigo Fonseca Lima, Rafael Santos Santana

**Affiliations:** ^1^ Department of Pharmacy, Faculty of Health Sciences University of Brasília Brasília Federal District Brazil

**Keywords:** clinical guidelines, community pharmacy, evidence‐based practice, minor ailments, pharmacist consultation

## Abstract

**Background:**

Minor ailments account for a substantial proportion of demands in community pharmacies, particularly in contexts marked by high self‐medication and risk of inappropriate medication use.

**Objective:**

To evaluate the adherence of pharmacological and non‐pharmacological interventions to clinical guidelines for minor ailment management and to analyze patient‐reported clinical outcomes.

**Methods:**

This observational, quantitative, descriptive study analyzed 227 pharmacist consultations. Interventions were individually reviewed, classified according to the GRADE system, and categorized according to adherence to pharmaceutical care guidelines. One week after the consultation, patients completed a questionnaire on clinical evolution.

**Results:**

A total of 332 pharmacological and 549 non‐pharmacological interventions were identified, with guideline adherence rates of 74.4% and 73.4%, respectively. The best performance was observed for Women's Health conditions, whereas gastrointestinal conditions showed the lowest adherence rates and the highest proportion of low‐quality evidence. Among 148 patients followed up, 75.7% reported complete symptom resolution and 24.3% partial improvement.

**Conclusions:**

The use of clinical guidelines contributed to more consistent interventions and favorable clinical outcomes, although challenges remain regarding gastrointestinal conditions and the standardization of pharmaceutical practice.

## 1 Introduction

Minor ailments account for a substantial proportion of healthcare demands managed in community pharmacies, particularly in settings characterized by high rates of self‐medication and direct patient demand for pharmacists' advice [1, 2]. In this context, community pharmacies play a strategic role in promoting the rational use of medicines, providing health education, and preventing complications associated with the inappropriate management of low‐complexity symptoms. Studies have shown that a large proportion of these conditions are managed outside traditional medical services, highlighting the pharmacist's role as a first point of contact within the healthcare system [3, 4].

The expansion of clinical pharmacy services in Brazil has extended pharmacists' role beyond medication dispensing to include clinical care, therapeutic counseling, and support for self‐care [3]. Federal Pharmacy Council (CFF) Resolutions No. 585/2013 and No. 586/2013 strengthened this role by formally recognizing pharmacists' clinical responsibilities and establishing that their interventions should be based on the best available scientific evidence [5, 6]. In this context, structured pharmacist interventions have been associated with improved clinical outcomes, reduced medication‐related problems, and enhanced patient safety [7, 8].

Despite these advances, clinical pharmacy practice in Brazilian community pharmacies remains highly heterogeneous. National studies have shown that, although pharmacists perform important clinical activities, challenges persist regarding the standardization of practice, the use of structured clinical protocols, the documentation of interventions, and the systematic evaluation of clinical outcomes [9, 10]. These limitations hinder the assessment of the effectiveness of pharmacy services and contribute to variability in the management of minor ailments.

In this context, evidence‐based practice (EBP) and the use of structured clinical guidelines are essential tools for improving pharmaceutical care. Methodologies such as the Grading of Recommendations Assessment, Development and Evaluation (GRADE) approach enable the assessment of the quality of evidence and the strength of clinical recommendations, supporting safer, more consistent, and reproducible therapeutic decision‐making [11, 12]. Furthermore, international experiences with structured minor ailment management services have demonstrated that the implementation of clinical protocols is associated with greater consistency of pharmacist interventions, improved clinical outcomes, and more appropriate use of healthcare services [4, 13, 14, 15].

Despite the expansion of clinical pharmacy services in Brazil, evidence simultaneously evaluating the adherence of pharmacist interventions to clinical guidelines and the clinical outcomes achieved by patients receiving care for minor ailments remains limited. Generating evidence in this context is essential to support the improvement of pharmacy services, strengthen evidence‐based practice, and demonstrate the clinical impact of pharmacist interventions in community pharmacies [9, 10].

Therefore, this study aimed to evaluate the adherence to pharmacological and non‐pharmacological interventions to the clinical guidelines used for the management of minor ailments and to assess patient‐reported clinical outcomes following pharmacist consultations.

## 2 Methods

### 2.1 Study Design

This was an observational, quantitative, descriptive‐analytical study based on a secondary analysis of records from pharmacist consultations conducted in community pharmacies between August 2024 and December 2025, together with information obtained through patient follow‐up after the pharmacist consultation.

### 2.2 Participants and Eligibility Criteria

The study included licensed pharmacists registered with the Regional Pharmacy Council (CRF) and final‐year undergraduate pharmacy students who participated in the Evidence‐Based Pharmacy course between 2024 and 2025. As part of the course, pharmacists provided consultations in community pharmacies using a standardized clinical documentation form. Pharmacy students conducted the consultations under the supervision of their preceptors.

Eligible records were required to be complete and legible, with a clear association between the patient's presenting complaint and the interventions performed. Records that were incomplete, illegible, did not comply with the required documentation format, or contained inconsistencies that precluded proper data interpretation and categorization were excluded from the analysis.

For the primary analysis, only records related to the clinical conditions covered by the guidelines used during the training program were included. Records concerning conditions not addressed in the course were excluded.

#### 2.2.1 Training Program and Clinical Guidelines

Training activities were conducted as part of the Evidence‐Based Pharmacy course. The curriculum covered minor ailments commonly managed in community pharmacies, including fever, headache, heartburn/dyspepsia, constipation, dermatological conditions, acute cough, and dysmenorrhea, with the aim of supporting evidence‐based management of these conditions.

The clinical guidelines used during the training program and for the evaluation of pharmacist interventions were extracted from the book Evidence‐Based Pharmacy: Pharmaceutical Care Guidelines for Minor Ailments, edited by Santana and Reis. The book compiles therapeutic recommendations developed through structured literature reviews and incorporates evidence from randomized controlled trials, observational studies, systematic reviews, and meta‐analyses. Evidence synthesis followed the Grading of Recommendations Assessment, Development and Evaluation (GRADE) approach, which classifies the quality of evidence and the strength of clinical recommendations [16].

In addition to recommendations developed for the Brazilian context, the book incorporates international protocols, including IndicaPro (Spain) and the NHS England Your Pharmacy First program [17, 18]. These clinical guidelines served as the educational framework for the training program and as the reference standard for evaluating the interventions recorded by participants throughout the study.

### 2.3 Data Collection

Data were obtained from pharmacist consultation records completed during the practical activities of the course and submitted through an electronic form (Google Forms). The standardized data collection form included information on the patient's presenting complaint, pharmacological and non‐pharmacological interventions, referrals, and the care plan.

One week after the consultation, patients who had received care from course participants completed a follow‐up questionnaire to assess their perceptions of clinical improvement and the effectiveness of the pharmacist consultation. The follow‐up questionnaire was developed by the research team, reviewed by five pharmacists, and pretested with 10 participants before its application. It was completed by the patients themselves and included the same questions for all conditions.

### 2.4 Variables

Clinical interventions were classified as pharmacological interventions, non‐pharmacological interventions, or referrals. Pharmacological interventions included medication dispensing or counseling regarding medication use.

Non‐pharmacological interventions comprised recommendations related to lifestyle modifications, hydration, diet, hygiene, and other self‐care measures. Referrals were defined as recommendations for evaluation at another healthcare service due to the presence of alarm signs, persistent symptoms, or the need for further investigation, in accordance with the clinical guidelines adopted in this study [16].

The classification of interventions was performed manually and independently reviewed by two evaluators. In cases of disagreement, a third evaluator was consulted to reach consensus.

Clinical outcomes were determined based on responses to the follow‐up questionnaire administered 1 week after the pharmacist consultation. For analytical purposes, outcomes were categorized into four groups: complete resolution, partial improvement, no improvement, and clinical worsening. Records without follow‐up questionnaire responses were retained in the database but excluded from the analysis of clinical outcomes.

### 2.5 Data Management and Statistical Analysis

Pharmacist consultation records were collected through an electronic form (Google Forms) and entered into Microsoft Excel for data management and organization. Statistical analyses were performed using Jamovi software. Records were organized according to the patient's presenting complaint, and the corresponding clinical interventions performed by the participants were identified.

For analytical purposes, minor ailments were grouped into five thematic categories (general, women's health, gastrointestinal, dermatological, and respiratory) based on their clinical similarities and the nature of the interventions (Table 1).

**Table 1:** Classification of the minor ailments covered by the clinical guidelines into thematic categories.

Thematic category	Minor ailments
General	Fever, headache, insomnia, and tobacco cessation
Women's health	Dysmenorrhea and emergency contraception
Gastrointestinal	Heartburn/dyspepsia, constipation, acute diarrhea, and nausea/vomiting
Dermatological	Acne vulgaris, pediculosis, onychomycosis, dermatomycoses, herpes labialis, superficial burns, and recurrent aphthous stomatitis
Respiratory	COVID‐19, common cold, laryngitis/pharyngitis, allergic rhinitis/sinusitis, and acute cough

Initially, the interventions were quantified by calculating the absolute frequency of each pharmacological and non‐pharmacological intervention according to the presenting complaint. Subsequently, each consolidated intervention was evaluated according to the quality of evidence and strength of recommendation based on the GRADE approach adopted in the clinical guidelines [16]. Interventions consistent with the corresponding clinical guidelines were classified as guideline‐adherent.

Based on these consolidated interventions, separate tables were developed for pharmacological and non‐pharmacological interventions, organized according to the minor ailment groups and accompanied by their respective frequencies. Descriptive analyses were performed by calculating absolute (N) and relative (%) frequencies to evaluate the alignment of interventions with the corresponding clinical guideline recommendations.

Subsequently, responses to the follow‐up questionnaires were analyzed to assess patient‐reported clinical outcomes. A table was constructed summarizing the number of records for each minor ailment group and their corresponding classifications according to clinical outcome.

Associations between minor ailment groups and the categorical variables were evaluated using Pearson's chi‐square test. When statistically significant associations were identified, adjusted standardized residuals were examined to determine which categories contributed most to the observed differences. The strength of associations was estimated using Cramér's V. Statistical significance was set at *p* < 0.05.

### 2.6 Ethical Considerations

This study was approved by the Research Ethics Committee in accordance with Brazilian National Health Council Resolution No. 466/2012, under approval number 5.171.224. All participants provided electronic informed consent by accepting the Informed Consent Form (ICF) before participation.

Participants' personal data were kept confidential and securely discarded upon completion of the study. The study protocol was registered in the Brazilian Registry of Clinical Trials (ReBEC) under registration number RBR‐6x8bx3y.

## 3 Results

### 3.1 Sample Characteristics

A total of 275 pharmacist consultation records were received. After applying the eligibility criteria, the final sample comprised 227 valid records, including consultations conducted by 100 undergraduate pharmacy students under the supervision of their preceptors and 69 licensed pharmacists.

### 3.2 Pharmacological Interventions

Table 2 presents the distribution of pharmacological interventions according to minor ailment group, together with their classification based on the quality of evidence and strength of recommendation established in the clinical guidelines.

**Table 2:** Pharmacological interventions by minor ailment group.

			Quality of evidence (GRADE)		Strength of recommendation (GRADE)	
Minor ailment group	*N*	Guideline adherence	High or moderate	Low, very low, or no evidence	Strong	Weak or no recommendation
General	57	49 (86.0%)	47 (82.5%)	10 (17.5%)	48 (84.2%)	9 (15.8%)
Women's health	40	36 (90.0%)	33 (82.5%)	7 (17.5%)	33 (82.5%)	7 (17.5%)
Gastrointestinal	69	43 (62.3%)	29 (42.0%)	40 (58.0%)	38 (55.1%)	31 (44.9%)
Dermatological	46	39 (85.0%)	40 (87.0%)	6 (13.0%)	36 (78.3%)	10 (21.7%)
Respiratory	120	80 (66.6%)	79 (66.0%)	41 (34.0%)	79 (66.0%)	41 (34.0%)
Total	**332**	**247 (74.4%)**	**228 (68.7%)**	**104 (31.3%)**	**234 (70.5%)**	**98 (29.5%)**

Abbreviation: GRADE, Grading of Recommendations Assessment, Development and Evaluation.

A total of 332 pharmacological interventions recorded during the consultations were analyzed. Overall, a high level of guideline adherence was observed, with most interventions supported by high‐ or moderate‐quality evidence and a predominance of strong recommendations **(**Table 2 **)**.

When comparing the different minor ailment groups, a significant association was found between the group evaluated and guideline adherence (*χ* ^2^ = 20.8; df = 4; *p* < 0.001; Cramér's V = 0.250). The *General* and *Women's health* groups showed higher‐than‐expected adherence, whereas the *Gastrointestinal* and *Respiratory* groups showed lower adherence.

Even greater differences were observed regarding the quality of evidence supporting the interventions (*χ* ^2^ = 39.0; df = 4; *p *< 0.001; Cramér's V = 0.343). The *Gastrointestinal* group showed the highest proportion of interventions supported by low‐, very low‐, or no‐quality evidence, whereas the *General*, *Women's health*, and *Dermatological* groups were predominantly supported by high‐ or moderate‐quality evidence.

The strength of recommendations also differed significantly among the minor ailment groups (*χ* ^2^ = 18.4; df = 4; *p* = 0.001; Cramér's V = 0.235). Again, the Gastrointestinal group presented the highest frequency of weak or no recommendations, whereas the General group showed the highest frequency of strong recommendations.

The most frequently reported pharmacological interventions are presented in Figure 1.

**Figure 1:**
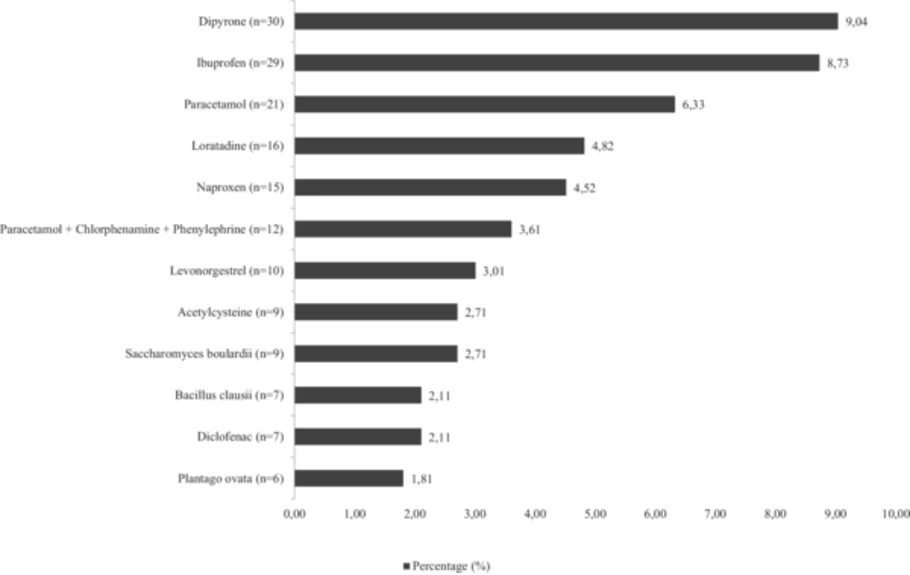
Most frequently reported pharmacological interventions.

The pharmacological interventions recorded largely reflected the management of minor ailments using over‐the‐counter (OTC) medicines, which have well‐established safety profiles [19]. The most frequently recommended therapeutic classes included analgesics and antipyretics (dipyrone, paracetamol), nonsteroidal anti‐inflammatory drugs (ibuprofen, naproxen), antihistamines (loratadine), and probiotics (*Saccharomyces boulardii* and *Bacillus clausii*).

### 3.3 Non‐Pharmacological Interventions

Table 3 presents the distribution of non‐pharmacological interventions according to minor ailment group, together with their classification based on the quality of evidence and strength of recommendation established in the clinical guidelines.

**Table 3:** Non‐pharmacological interventions by minor ailment group.

			Quality of evidence (GRADE)		Strength of recommendation (GRADE)	
Minor ailment group	*N*	Guideline adherence	High or moderate	Low, very low, or no evidence	Strong	Weak or no recommendation
General	124	87 (70.1%)	29 (23.4%)	95 (76.6%)	88 (71.0%)	36 (29.0%)
Women's health	67	57 (85.1%)	43 (64.2%)	24 (35.8%)	41 (61.2%)	26 (38.8%)
Gastrointestinal	112	80 (71.4%)	48 (42.9%)	64 (57.1%)	80 (71.4%)	32 (28.6%)
Dermatological	70	49 (70.0%)	37 (52.9%)	33 (47.1%)	34 (48.6%)	36 (51.4%)
Respiratory	176	130 (73.9%)	95 (54.0%)	81 (46.0%)	119 (67.6%)	57 (32.4%)
Total	**549**	**403 (73.4%)**	**252 (45.9%)**	**297 (54.1%)**	**362 (66.0%)**	**187 (34.0%)**

Abbreviation: GRADE, Grading of Recommendations Assessment, Development and Evaluation.

A total of 549 non‐pharmacological interventions recorded during the consultations were analyzed. Overall, a high level of guideline adherence was observed, although most recommendations were supported by low‐, very low‐, or no‐quality evidence (Table 3).

No significant association was observed between minor ailment group and guideline adherence (*χ* ^2^ = 6.00; df = 4; *p* = 0.199; Cramér's V = 0.105).

In contrast, a significant association was found between minor ailment group and the quality of evidence supporting non‐pharmacological interventions (*χ* ^2^ = 40.7; df = 4; *p* < 0.001; Cramér's V = 0.272). The *Women's health* and *Respiratory* groups showed a higher‐than‐expected frequency of interventions supported by high‐ or moderate‐quality evidence, whereas the *General* group showed a higher‐than‐expected frequency of interventions supported by low‐, very low‐, or no‐quality evidence.

A significant association was also observed between minor ailment group and the strength of recommendations (*χ* ^2^ = 13.2; df = 4; *p* = 0.010; Cramér's V = 0.155). The Dermatological group showed a higher‐than‐expected frequency of weak or no recommendations.

The most frequently reported non‐pharmacological interventions are presented in Figure 2.

**Figure 2:**
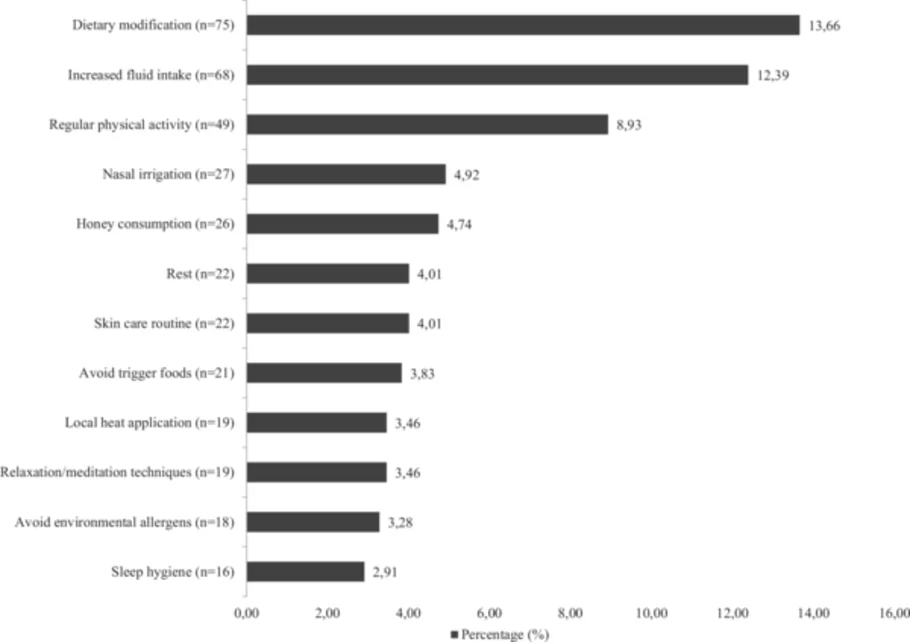
Most frequently reported non‐pharmacological interventions.

It was observed that dietary modification (13.7%), increased fluid intake (12.4%), and regular physical activity (8.9%) were the most frequently reported recommendations, followed by simple symptom relief or preventive measures, such as nasal irrigation, rest, and local heat application.

Recommendations to avoid trigger foods were also identified, particularly for headache, a condition included in the General group, and were related to the identification and avoidance of potential triggering factors, such as caffeine, chocolate, processed meats, and alcoholic beverages.

### 3.4 Referrals

Regarding the need for continuity of care in other healthcare settings, 11 referrals (4.8%) were recorded among the 227 consultations analyzed, including 3 in the General group, 1 in the Women's Health group, 3 in the Gastrointestinal group, 1 in the Dermatological group, and 3 in the Respiratory group.

### 3.5 Patient‐Reported Clinical Outcomes

Table 4 presents the patient‐reported clinical outcomes 1 week after the pharmacist consultation, stratified by minor ailment group. The results reflect the proportion of patients who reported complete or partial symptom resolution, providing an assessment of the perceived effectiveness of the interventions performed.

**Table 4:** Patient‐reported clinical outcomes.

Minor ailment group	Records	Respondents	Complete resolution	Partial improvement
General	54	39 (72.2%)	31 (79.5%)	8 (20.5%)
Women's health	28	15 (53.6%)	14 (93.3%)	1 (6.6%)
Gastrointestinal	46	31 (67.4%)	25 (80.6%)	6 (19.4%)
Dermatological	33	20 (60.6%)	11 (55.0%)	9 (45.0%)
Respiratory	66	43 (65.2%)	31 (72.1%)	12 (27.9%)
Total	**227**	**148 (65.2%)**	**112 (75.7%)**	**36 (24.3%)**

Of the 227 records evaluated, 148 (65.2%) had a completed follow‐up questionnaire 1 week after the pharmacist consultation. Among these respondents, 112 (75.7%) reported complete symptom resolution, whereas 36 (24.3%) reported partial improvement, with no cases of clinical worsening.

The follow‐up response rate did not differ significantly across minor ailment groups (*χ* ^2^ = 3.25; df = 4; *p* = 0.517; Cramér's V = 0.120), indicating a homogeneous distribution of respondents among the groups.

Although differences were observed in the proportion of complete symptom resolution across minor ailment groups, no statistically significant association was found between the group evaluated and the patient‐reported clinical outcome (*χ* ^2^ = 8.21; df = 4; *p* = 0.084; Cramér's V = 0.236). Overall, most participants reported complete symptom resolution following the pharmacist consultation, suggesting favorable clinical outcomes regardless of the minor ailment group evaluated.

## 4 Discussion

The profile of the interventions recorded in this study is consistent with patterns reported internationally for the management of minor ailments. Pharmacological interventions were predominantly composed of analgesics, antipyretics, nonsteroidal anti‐inflammatory drugs (NSAIDs), and antihistamines, a pattern consistent with medication use in Brazilian primary care, where analgesics and antipyretics are the most commonly used medications for minor health conditions [20, 21]. Studies conducted in the United Kingdom and Canada have similarly shown that these medications account for most recommendations in structured minor ailment services, reflecting their well‐established use, proven effectiveness, and favorable safety profiles [19, 22, 23].

The recommendation of levonorgestrel, probiotics, and acetylcysteine, although less frequent, reflects common demands in Brazilian community pharmacy practice and is consistent with the clinical guidelines adopted during the training program, which address conditions such as emergency contraception, mild dyspepsia, constipation, and acute cough [16, 22].

Regarding non‐pharmacological interventions, recommendations such as increased fluid intake, dietary modification, and regular physical activity were frequently reported, although these measures are not always explicitly addressed in the clinical guidelines, which prioritize more condition‐specific recommendations. Nevertheless, this pattern is consistent with the expanded role of pharmacists in promoting self‐care and aligns with World Health Organization (WHO) recommendations, which emphasize health education and lifestyle modification as key strategies for preventing chronic diseases and managing minor health conditions [16, 24].

The evaluation of pharmacist interventions demonstrated a high level of adherence to the clinical guidelines, particularly for pharmacological interventions, with 74.4% of recommendations classified as guideline‐adherent. A significant association was observed between minor ailment groups and guideline adherence, with the Women's Health and General groups showing higher‐than‐expected adherence rates. In contrast, the Gastrointestinal and Respiratory groups exhibited lower‐than‐expected adherence.

Regarding the quality of evidence, the Women's Health group had the highest proportion of interventions supported by high‐ or moderate‐quality evidence, whereas the Gastrointestinal group showed the highest frequency of interventions supported by low‐ or very low‐quality evidence. This finding may reflect the inherent complexity of gastrointestinal conditions, which often require differentiation between functional and organic disorders, identification of alarm signs, and, in many cases, management of nonspecific symptoms [13, 16].

Regarding non‐pharmacological interventions, no significant differences in guideline adherence were observed among the minor ailment groups. However, a significant association was found between minor ailment group and the quality of evidence supporting the recommendations, with the Women's Health and Respiratory groups showing a higher frequency of interventions supported by high‐ or moderate‐quality evidence. In addition, the Dermatological group exhibited a higher frequency of recommendations classified as weak or unsupported by evidence.

The literature describes the management of dermatological conditions in community pharmacies as an area characterized by greater variability in clinical practice, the need for more advanced clinical judgment, and gaps in pharmacists' specialized dermatology training. Dermatological conditions often require more detailed and long‐term self‐care recommendations, with outcomes strongly dependent on patient adherence, which may make non‐pharmacological interventions more difficult to implement and standardize in routine practice [25, 26].

The differences observed among the minor ailment groups appear to reflect the interplay between the available evidence, the degree of protocol standardization, and healthcare professionals' familiarity with each condition. The higher level of guideline adherence observed in the Women's Health group may also be explained by the greater clarity and maturity of the recommendations available for these conditions. Clinical guidelines for dysmenorrhea and emergency contraception are widely disseminated by organizations such as the Brazilian Federation of Gynecology and Obstetrics Associations (FEBRASGO) and the World Health Organization (WHO). In addition, recent regulations, such as Federal Pharmacy Council (CFF) Resolution No. 12/2024, have formally expanded pharmacists' clinical scope of practice in this field [27, 28, 29].

International studies have shown that adherence to clinical protocols tends to be higher for conditions supported by more objective and well‐established recommendations, such as those related to Women's Health [13, 27]. Conversely, conditions characterized by more diffuse and overlapping symptoms, including dyspepsia, constipation, and nausea, are associated with greater variability in clinical interpretation. In the case of dermatological conditions, the reliance on visual assessment and the morphological diversity of skin lesions may have contributed to the greater variability in clinical decision‐making and the lower consistency of interventions observed in the present study [13, 16].

Among non‐pharmacological interventions, recommendations were also predominantly supported by low‐ or very low‐quality evidence, which is consistent with the application of the GRADE approach to behavioral and self‐care interventions. These interventions are often supported by studies with small sample sizes, lack of blinding, and considerable variability in intervention protocols, all of which reduce the certainty of the available evidence. Nevertheless, the GRADE approach allows strong recommendations to be made even when the certainty of evidence is limited, provided that the balance between benefits, risks, and costs is favorable [11, 12, 16].

In addition to these clinical and methodological aspects, factors beyond the clinical protocols may also contribute to the variability observed in pharmacists' interventions. Patient preferences and beliefs, previous experiences with medications, socioeconomic conditions, and access to healthcare services and health products all play an important role in shaping pharmacists' therapeutic decisions. These determinants are particularly relevant in the Brazilian context, where structural barriers to healthcare access often require adaptation of guideline recommendations to meet the individual needs of each patient [16, 30, 31].

Furthermore, in the Brazilian context, studies such as that by Garabeli et al. have shown that most studies evaluating pharmacy practice fail to clearly report intervention indicators and frequently lack standardized clinical protocols. Therefore, although the proportion of guideline‐adherent interventions observed in the present study can be considered relatively high compared with the heterogeneity previously reported, the findings still indicate opportunities for improvement, particularly in minor ailment groups with lower levels of guideline adherence [9].

Regarding clinical outcomes, complete symptom resolution was reported in 75.7% of cases, whereas 24.3% of patients reported partial improvement, with no cases of clinical worsening. The high rate of symptom resolution observed in the present study is consistent with findings from structured pharmacist‐led minor ailment services, such as the Spanish INDICA + PRO program, in which the proportion of patients reporting symptom improvement ranges from approximately 68% to 94%. Within this program, the combination of guided self‐care, structured triage protocols, the use of clinical guidelines, and appropriate referral of more complex cases was associated not only with improved clinical outcomes but also with more efficient use of healthcare resources [17].

Although numerical differences in clinical outcomes were observed among the minor ailment groups, no statistically significant association was found between minor ailment group and patient‐reported clinical outcomes. Nevertheless, the Dermatological group showed the lowest proportion of complete symptom resolution and the highest frequency of partial improvement. This pattern may be related to the clinical course of certain self‐limiting dermatological conditions, which often require longer periods for symptom resolution and sustained adherence to self‐care measures. In contrast, the Gastrointestinal group showed a high rate of complete symptom resolution despite the lower level of adherence to pharmacological guidelines, suggesting that additional clinical and patient‐related factors may also influence the course of these conditions.

The analysis of referrals to other healthcare services further supports the safety of this care model. Referrals were more frequent among the Gastrointestinal, Respiratory, and Dermatological groups, suggesting that although most minor ailments can be effectively managed in community pharmacies, some cases require further medical evaluation. These findings are consistent with previous studies highlighting the pharmacist's role as a first point of contact within the healthcare system, with the ability to identify patients requiring referral for specialized care [16, 17].

From an impact perspective, the findings of the present study are consistent with the international literature on structured minor ailment services in community pharmacies. Clinical trials evaluating these services have shown that protocol‐based models combined with systematic follow‐up improve the appropriateness of pharmacist interventions, increase symptom resolution, and enhance patient‐reported quality of life compared with usual care, with symptom resolution rates exceeding 85%–90% and improvements in short‐term well‐being scores [32, 33].

Similarly, the high proportion of complete symptom resolution and partial improvement observed in the present study reinforces that pharmacist consultations supported by clinical guidelines can achieve favorable clinical outcomes in the management of minor ailments. These findings are consistent with evidence demonstrating that community pharmacies can serve as effective first points of contact within the healthcare system [33]. Beyond their clinical and humanistic benefits, economic evaluations have shown that structured pharmacist‐led minor ailment services, including prescribing when appropriate, are cost‐effective or generate direct savings for healthcare systems by reducing physician consultations and the use of higher‐complexity healthcare services, as demonstrated in cost‐utility analyses conducted in Spain and cost‐minimization models developed in Canada [34, 35].

Taken together, the findings of the present study suggest that incorporating clinical guidelines into routine community pharmacy practice may simultaneously improve clinical outcomes, enhance patient experience, and promote a more efficient use of healthcare resources.

## 5 Study Limitations

This study has some limitations. Patient‐reported clinical outcomes are subject to recall bias and loss to follow‐up. In addition, the inclusion of both undergraduate pharmacy students and licensed pharmacists may have introduced heterogeneity in the level of detail of the consultation records and the documentation of pharmacist interventions. Furthermore, the follow‐up questionnaire used the same questions across all conditions and did not include a standardized measurement of symptom severity before and after the consultation, which limits the comparability of outcomes among minor ailment groups.

## 6 Conclusion

The present study demonstrated that the use of structured clinical guidelines can enhance pharmacist consultations for the management of minor ailments, highlighting the applicability of standardized protocols in community pharmacy practice. The recorded interventions showed good adherence to clinical guideline recommendations for both pharmacological and non‐pharmacological interventions, reinforcing the value of these tools in supporting safe, evidence‐based, and patient‐centered clinical decision‐making, as well as the importance of continuous professional training and education.

The differences observed among the minor ailment groups suggest that condition‐specific clinical characteristics influence the implementation of guideline recommendations and the quality of pharmacist interventions. Women's Health conditions showed the highest level of guideline adherence, whereas Gastrointestinal and Dermatological conditions exhibited greater variability across some of the evaluated indicators. Nevertheless, the high proportion of complete symptom resolution and partial improvement highlights the potential of protocol‐supported pharmacist consultations to achieve favorable clinical outcomes in the management of minor ailments.

Furthermore, the high proportion of non‐pharmacological interventions supported by low‐certainty evidence underscores the need for further research on self‐care interventions and non‐pharmacological strategies used in routine clinical practice.

Overall, these findings reinforce the role of clinical guidelines as important tools for improving the standardization, safety, and quality of pharmaceutical care. Enhancing clinical documentation, promoting continuous professional training, and strengthening the integration of scientific evidence into routine pharmacy practice represent important strategies for improving the effectiveness of pharmacy services and supporting safe, effective, and evidence‐based self‐care.

## Author Contributions

**Letícia Isabella Ferreira Brandão:** methodology, investigation, data curation, formal analysis, visualization, writing – original draft. **Gustavo Pereira Calado:** methodology, investigation, data curation, writing – review and editing. **Laura Beatriz Gomes Brandão:** methodology, supervision, writing – review and editing. **Maria Christina dos Santos Verdam:** investigation, writing – review and editing. **Débora Santos Lula Barros:** writing – review and editing. **Rodrigo Fonseca Lima:** writing – review and editing. **Rafael Santos Santana:** conceptualization, methodology, investigation, supervision, project administration, writing – review and editing.

## Funding

The authors have nothing to report.

## Ethics Statement

The study was approved by the Research Ethics Committee under approval number 5.171.224 and registered in the Brazilian Registry of Clinical Trials (ReBEC) under identifier RBR‐6x8bx3y.

## Conflicts of Interest

The authors declare no conflicts of interest.

## Data Availability

The data that support the findings of this study are available from the corresponding author upon reasonable request.
